# Mumps in the Vaccination Age: Global Epidemiology and the Situation in Germany

**DOI:** 10.3390/ijerph15081618

**Published:** 2018-07-31

**Authors:** Andrea-Ioana Beleni, Stefan Borgmann

**Affiliations:** 1 Department of Urology and Comprehensive Cancer Center, Vienna General Hospital, Medical University of Vienna, Währinger Gürtel 18-20, 1090 Vienna, Austria; andrea-ioana.beleni@meduniwien.ac.at; 2 Department of Infectious Diseases and Infection Control, Hospital of Ingolstadt, D-85049 Ingolstadt, Germany

**Keywords:** Jeryl Lynn, Leningrad-Zagreb, measles-mumps-rubella vaccination, orchitis, Urabe, vaccination effectiveness, waning immunity

## Abstract

Vaccination against mumps virus (MuV) (mostly measles-mumps-rubella) is routinely performed in more than 120 countries and has resulted in a distinct decrease of mumps incidence. However, alteration of mumps epidemiology has been observed in several countries after implementation of the vaccine but is sparsely documented. Moreover, outbreaks have occurred after starting vaccination, even in highly vaccinated populations. In the former German Democratic Republic (DDR) mumps was a notifiable disease but vaccination against mumps was not implemented. In the five eastern German states forming the DDR until 1990, mumps was not notifiable until 2001. Except for the lack of reporting between 1990–2000, data from Eastern Germany allow analysis of mumps epidemiology after initiating the vaccination campaign. For the period from 2001 to 2016 the data show that the incidence of mumps dropped notably after initiating vaccines, and was accompanied by an increase of the median age of patients with mumps. In Eastern Germany, no outbreaks were noted, while several outbreaks occurred in Western Germany, possibly due to a lower vaccination rate. Further literature analysis revealed that outbreaks were facilitated by waning immunity and crowding. Nevertheless, although vaccination prevented infection, the course of illness, once infected, was sometimes more complicated. In comparison to non-vaccinated populations, high rates of complicated courses occurred and were marked by orchitis, due to higher age of mumps patients. Therefore, refusing vaccination against mumps increases the risk of severe courses when living in a vaccinated population.

## 1. Introduction

Mumps is characterized by an inflammation of the salivary glands, resulting in painful swelling of one or both parotic glands. Complicated courses may result in meningitis, encephalitis, pancreatitis, and hearing defects. Female patients may also develop oophoritis, while male patients can develop orchitis.

Like other childhood diseases, the severity of the disease increases with age. About one third of mumps virus (MuV) infections are asymptomatic [1] and small children often exhibit no symptoms [2]. Males exhibit a markedly higher risk of suffering from complications at higher age than females [3]. The probability of developing orchitis was calculated to be 25% for post-puberty males [1,4]. In vaccinated populations, the percentage of asymptomatic infections is high even in older patients. For example, in an outbreak of predominately vaccinated Dutch students about two thirds of infections were asymptomatic [1,5]. MuV is not a tenacious virus particle and has a low transmission factor. Before vaccination was available, incidence of mumps was 100–1000 pro 100,000 persons per year, exhibiting epidemic peaks every 2–5 years. In the temperate zone most infections occurred in winter and spring while no seasonality was observed in the tropics [6].

## 2. The Mumps Virus and Its Genotypes

The MuV was considered to infect only humans. However, some years ago virus particles with the potential to infect humans were discovered in the spleen of a bat [7,8].

**Figure 1:**
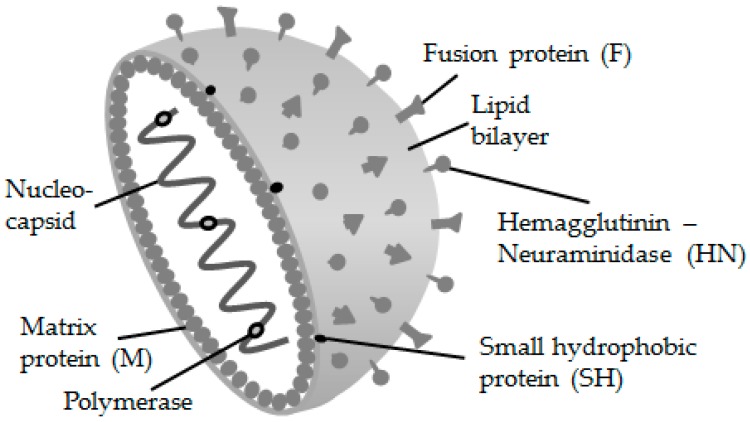
Structure of the mumps virus. The helical nucleocapsid consists of the nucleoprotein and RNA.

The genome of the MuV is a single-stranded RNA exhibiting 15,384 nucleotides encoding 12 proteins [6]. The core of the MuV is surrounded by an envelope (Figure 1). A glycoprotein called mumps hemagglutinin-neuraminidase (HN) and the fusion protein (F) decorate the envelope and form spikes visible with an electron-microscope. Although antibodies to various proteins are generated, only those targeting NH mediate protection [9].

When comparing the nucleotide sequences of various mumps viruses, the sequence of the small hydrophobic protein (SH) shows highest diversity. Therefore, nucleotide sequences of the SH gene are used to categorize MuV into various genotypes [10]. Currently, 12 genotype groups are present named with the capital letters A–N. Genotypes E and M are omitted because MuV previously belonging to these groups were later assigned to other groups. Furthermore, there are some MuV not indexed yet. Some genotypes were further divided in certain subgroups. Genotypes A and B were already identified in the 1960’s. A prominent strain belonging to genotype A is the Jeryl Lynn MuV (JL). Two closely related JL subtypes (JL2, JL5) are still used for the production of a vaccine. Another MuV used for vaccine production is Urabe AM9 belonging to genotype B. Genotype A and B viruses were frequently isolated in Europe and in Japan, respectively. However, wild type MuV belonging to these genotypes did not occur since the 1990s. Until 2012 MuV from 38 countries had been isolated. According to the results of these analyses genotypes C, D, H, and J were more prevalent in the Western hemisphere while genotypes B, F, G, and I were more common in Asian countries. However, additional genotypes can be imported and multiple genotypes may simultaneously circulate in a country relativizing the statement of global strain distribution [11]. Within the past years in various vaccinated populations outbreaks have occurred. Mostly, these outbreaks had been caused by the global genotype G. Apart from genotype G single outbreaks were caused by genotype H MuV. Recently, outbreaks were also caused by genotype K MuV in Eastern Asia [12].

The diversity of MuV is not reflected by the human antibody response. Human antibodies form a single serotype because the binding sites of the antibodies are very similar.

## 3. Vaccination against the MuV

Commonly, the measles-mumps-rubella vaccine (MMR) is used for vaccination. Before initiation of a vaccine program in the UK, Anderson et al. calculated that a vaccination rate of 60% will change mumps epidemiology extensively [3]. The incidence would be reduced and the period between epidemic peaks would be increased. The total number of mumps related case complications was predicted to decrease, while the number of patients suffering from mumps orchitis was expected to rise [3].

Mumps vaccine had been introduced nationwide in 121 countries belonging to the World Health Organization (WHO) by the end of 2016 [13]. However, in most of the African, South Asian and South-eastern Asian countries there is no sufficient coverage.

Initially, one dose of MMR vaccine was recommended. In many countries a second dose of the vaccine was added to the vaccination schedule later, in order to “optimize” immune-response, and, in particular, to increase the proportion of individuals exhibiting antibodies. However, a meta-analysis revealed that the second application only restores immunity up to the level of the first vaccine [14]. In the USA the first MMR vaccine is applied at the age of 12–15 months and the second vaccine at the age of 4–6 years. In Germany the first MMR vaccine is given at the age of 11–14 months and the second vaccine at the age of 15–23 months. If one or both vaccines are lacking an individual should receive a MMR vaccine at the age of 2–17 year. The reason for early vaccination in Germany is that children entering kindergarten or school will be fully vaccinated against measles.

Current vaccine schedules valid for countries of the European Union are available from the European Centers for Disease Control (ECDC) website [15].

As predicted by Anderson et al., incidence of mumps markedly decreased after vaccination campaigns had been started [3]. Unfortunately, after initiating vaccination campaign, mumps surveillance was performed only in a few countries. In the USA a 97% decrease was observed between 1968–1982 and in Finland the incidence was <1 pro 100,000 inhabitants per year [16].

Many different strains are theoretically available as the MuV component of the MMR. Application of various strains was regionally or nationally limited. Consequently, information about those strains, e.g., side effects and vaccine effectiveness, is limited. Examples of less characterized strains are Hoshino, Miyahara, Torii, and NK M-46 applied in Japan, the S-12 strain from Iran, and the Sofia-6 strain from Bulgaria. More extended information is available for the following vaccine strains: JL, Urabe, Rubini, Leningrad-3, and its derivate Leningrad-Zagreb. Except for Rubini, all strains are recommended by the WHO for vaccination against mumps. The Rubini strain was excluded because of insufficient vaccine effectiveness. This is shown in a review article determining the vaccine effectiveness (VE) after application of a single vaccine. In that study VE of the JL strain was 72.8–91%, that of the Urabe strain 54.4–93%, and that of the Rubini strain 0–33% [17]. Effectiveness of strains recommended by the WHO is up to 100%. However, this data were determined in non-outbreak situations. In an outbreak setting vaccine effectiveness is lower [18].

Currently, in Western Europe and the USA MMR vaccines contain either JL or a derivate strain of JL (e.g., RIT4385). The manufacturers argue that these strains cause lower rates of aseptic meningitis, the major side effect of mumps vaccine. Indeed, an association of mumps vaccine strains other than JL to aseptic meningitis recently has been described [19].

Variations in amino-acid sequences of MuV proteins have been discussed to facilitate the formation of aseptic meningitis. However, there was no association of certain polymorphisms in the NH and the SH gene with the development of this side effect [20].

In two Brazilian studies the number of doses of Leningrad-Zagreb strain applied per one case of aseptic meningitis ranged from 3390 to 19,247 [21,22]. In Slovenia two cases of aseptic meningitis occurred after having applied 100,000 doses Leningrad-Zagreb vaccine (one case per 50,000 vaccinations) while in Croatia that rate was markedly higher (90 cases/100,000 doses or one case per 1111 vaccinations) [11]. By contrast, aseptic meningitis was not observed in 453,119 Egyptian children after receiving MMR vaccine containing Leningrad-Zagreb MuV [23]. In Slovenia, the rate of aseptic meningitis was estimated to be 20–100 cases after receiving 100,000 doses Leningrad-3 vaccine [11].

The rate of aseptic meningitis was 0.1–1 case per 100,000 doses of the JL strain and, therefore, lower than those after applying the vaccine strains mentioned above [11]. On the other hand, JL and derivate strains are much more expensive than others (Table 1). Therefore, in developing countries mostly Urabe and Leningrad-3 strain or its derivates were used. The approval of these strains by the corresponding authorities is comprehensible, since the course of aseptic meningitis after vaccination is usually mild and VE seems to be similar to that of the JL strain [24].

However, due to high rates of aseptic meningitis, the approval of vaccines containing the Urabe strain was withdrawn in several countries and in 2015 production of the Urabe strain was stopped [26].

Application of JL vaccines may cause a higher rate of febrile seizures. This has been observed when the first vaccine had been applied at the age of 16 month or later.

In Germany the “Standing Committee for Vaccination” (STIKO) is the responsible authority for developing current vaccination recommendations. Recommended vaccinations are payed by the legal health insurance companies. Moreover, the Federal Republic of Germany pays the costs resulting from side effects of a vaccination. In Germany vaccines against mumps are available as the MMR and the MMR varicella (MMRV) vaccine (Table 1). In comparison to MMR, the MMRV vaccine is associated with a higher rate of febrile seizures [27,28]. Therefore, pediatricians often suggest simultaneous application of MMR and chicken pox vaccines. However, parents often disagree to pricking their children twice resulting in a lower vaccination quote for VZV vaccine in comparison to MMR [29]. Nevertheless, a sufficient coverage of MMRV vaccine would lead to a lower number of hospital admissions of children suffering from chicken pox, compared to a low number of hospitalizations resulting from MMRV vaccine leading to febrile seizures [30]. In contrast to the initial dose of vaccine, application of the second MMRV dose is not associated with a higher number of febrile seizures [31].

Within six weeks after being inoculated with MMR vaccine there is an increased risk to develop idiopathic thrombocytopenic purpura (ITP) [32]. The risk was estimated an ITP pro 40,000 MMR vaccines [33].

In a Cochrane analysis there was no association of MMR vaccine with the development of numerous diseases: autism, asthma, leukemia, hay fever, diabetes mellitus type 1, ataxia, M. Crohn, demyelinating diseases and viral infections [32]. In 1998, the association of the MMR vaccine with autism and M. Crohn had become a political issue. Referring to his current Lancet paper Andrew Wakefield (1998) postulated the association of the MMR vaccine to these diseases when giving public talks, although those associations had not been described in the publication. Twelve years later, the manuscript was retracted by all of his co-authors and the journal. Moreover, the association of the MMR vaccine to autism and M. Crohn was disproved [34,35]. Nevertheless, in 2014 the association of MMR vaccine with autism was postulated again [36]. However, eventually also that manuscript was retracted by the publisher “because of serious concerns about the validity of its conclusion” [37].

## 4. Outbreaks in the Vaccination Age

After vaccination campaigns had been initiated in various populations, mumps outbreaks occurred. Although excluded by the results of a German study [38], most authors identified waning immunity as the major reason for infection. This finding is plausible because vaccination caused antibody production in general is not as efficient as that induced by natural infection [39,40]. Waning immunity after vaccination might be explained by the finding that vaccination does not induce the generation of long lasting response mediated by memory T-cells. In contrast to childhood vaccination, MuV infection induced the formation of persistent polyclonal CD8^+^ memory T-cells [41]. Table 2 summarizes the results of studies examining waning immunity.

Although denied in a recent study, genetic mismatch of vaccine and wild type viruses was discussed to facilitate ongoing spread of MuV [14]. Recent outbreak viruses were predominantly associated with genotypes G, H, and K, while vaccine viruses belonged either to genotype A (JL) or genotype B (Urabe). Genotype of the Leningrad-Zagreb vaccine MuV had not been determined yet. To prevent mismatch of wild-type and vaccine strains, consideration whether the application of a polyclonal mumps vaccine to overcome that problem was discussed [54]. The importance of a genetic match of vaccine and wild-type MuV was demonstrated in a clever experiment [9]. In that experiment, inactivation capacity of antibodies against the JL MuV and a genotype H wild type virus were compared. As expected, antibodies against JL MuV more efficiently inactivated a JL virus, while antibodies against the genotype H virus more effectively inactivated the corresponding genotype H virus. Substituting the immunogenic HN gene of both MuV with that of the other MuV antibodies against the JL virus was shown to more efficiently inactivate the recombinant genotype H virus, while antibodies against genotype H wild-type virus more effectively inactivated the recombinant JL MuV. This substitution was not observed when genes encoding other proteins were swapped [9]. These results also confirm the observation that the HN is the most immunogenic protein of the MuV. From a medical view, gene sequence of NH might be more appropriate for categorization of MuV than the SN sequence.

In some outbreak countries vaccination rates were low, while in other countries high rates were observed [55,56,57,58]. In those populations, outbreaks were facilitated by close contacts, predominately between adolescents and young adults, e.g., between house-mates living in a commune and at student parties [59]. Infections were also observed where children resided closely together, for example at a holiday camp [60] and in a Jewish school where the pupils studied text for hours in a crowded environment [61].

**Figure 2:**
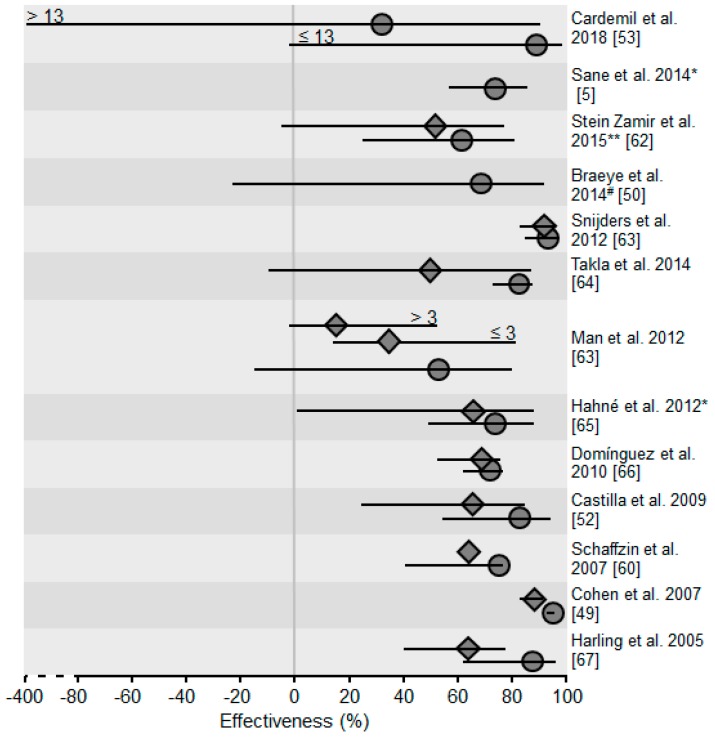
Vaccination effectiveness (%) after MMR-vaccination in published analyses. Studies were obtained by performing a “Pubmed” request [42] using the search terms “mumps” and “vaccination effectiveness” and “outbreak”. Data were accessed on 29 March 2018. Rhombuses: Effectiveness after 1 MMR dose. Circles: Effectiveness after 2 doses. Lines: 95%-Confidence intervalls. >13 = Effectiveness when second vaccine dose was >13 years ago. ≤13 = Effectiveness when second vaccine dose was ≤13 years ago. *^/^**: Effectiveness against orchitis * or other complcations **. ^#^: Effectiveness of 2 doses vs. 1 dose. ≤3 = Effectiveness when first vaccine dose was ≤3 years ago. >3 = Effectiveness when first vaccine dose was >3 years ago.

In a period without an outbreak, effectiveness of the mumps vaccine is estimated up to 100%. As shown in Figure 2, even in an outbreak situation vaccine protects against infection and from developing complications (e.g., orchitis) [5,49,50,52,53,60,62,63,64,65,66,67]. Furthermore, the risk of becoming hospitalized was reduced by about 50% when at least one dose had been received [61].

Application of a third MMR vaccine resulted in a significant increase of antibody titers against the JL vaccine virus. However, 12 months later titers were similar to those prevalent before the third vaccine [68]. Similar data were obtained in a recent analysis. However, in that study, individuals exhibiting low antibody titers before receiving the third vaccine [69] gained the most from revaccination. Therefore, a third vaccine is not generally recommended but seems helpful to limit an outbreak [15,54,70,71,72,73].

## 5. The Epidemiologic Situation in Germany

In 1990 the German Democratic Republic (DDR) on the eastern territory of Germany joined the Federal Republic of Germany (BRD) on the western territory. In the BRD vaccination against mumps was started in 1976. Mumps is a notifiable disease since 2013. Therefore, data about the situation in the Western part of Germany are rare. In the DDR mumps had been a notifiable disease since 1964 but after joining BRD reporting obligation was abolished. In 2001 mandated reporting based on regional state regulations was reintroduced in the five eastern states that formed the pre-existing DDR. In the eastern states (Eastern Germany) vaccination was started in 1991. Although there are no data available about the situation from 1991 to 2000 the late introduction of the vaccine in Eastern Germany allows an assessment of the impact of a vaccination campaign on the epidemiologic situation. The reported data in the DDR displayed the cyclic course of mumps incidence. The lowest incidence was observed in 1986 (155 infections/100,000 inhabitants) and the highest incidence in 1997 (1301 infections/100,000 inhabitants) [38]. After starting the vaccination campaign, incidence markedly decreased. In 2016, 0.62 infections/100,000 inhabitants were noticed.

**Figure 3:**
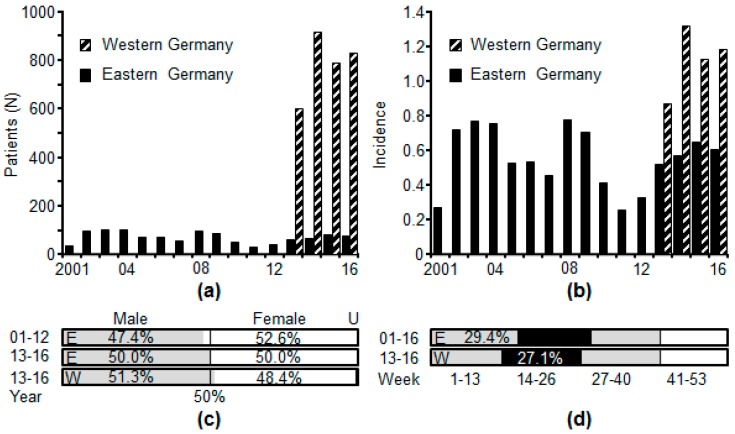
Notifications of mumps cases in Western and Eastern Germany 2001–2016. In Western Germany notification of mumps started in 2013 and in Eastern Germany in 2001. (**a**) Number of affected individuals. (**b**) Incidence (cases/100,000 persons). (**c**) Percentage of male and female patients. (**d**) Seasonal distributions of mumps notifications. E = Eastern Germany. W = Western Germany. U = Unknown sex. Data were obtained from the Survstat tool of the German Robert-Koch-Institute (RKI) [74]. Data were accessed on 12 April 2018. Eastern Germany comprised Brandenburg, Mecklenburg-Western Pomerania (Meckenburg-Vorpommern), Saxony, Saxony-Anhalt and Thuringa. The other 11 federal states including Berlin were regarded as Western Germany.

Figure 3 shows the incidence of mumps basing on reporting obligation data in Eastern (2010–2016) and Western Germany (2013–2016). Data were obtained from the “survstat” module, retrievable at the homepage of the Robert Koch-Institute (RKI) [74]. The figure shows that the incidence in Eastern Germany was lower than in the Western part. The lower incidence might have been caused by a higher vaccination rate in Eastern Germany. In the year 2000, the vaccination rate in Eastern Germany was 97.6% (first vaccine) and 92.7% (second vaccine) and in Western Germany 95.8% (first vaccine) and 91.0% (second vaccine) [38]. Although the difference is small it obviously has far reaching implications on herd immunity. As shown by Figure 3c percentage of male and female mumps patients was similar in both parts of the countries and most infections were noticed the cold seasons (Figure 3d).

**Figure 4:**
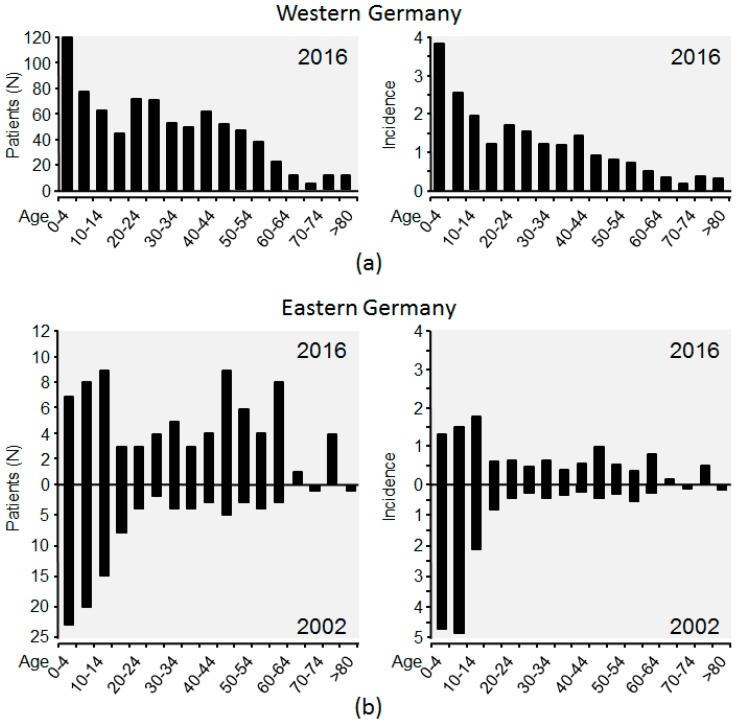
Age of notified patients suffering from mumps in Western and Eastern Germany. Age is given in interval of 5 years. (**a**) Age of affected patients in Western Germany in 2016. (**b**) Age of affected patients in Eastern Germany in 2002 and 2016. Left panels: Number of affected patients per 5-year interval. Rights panels: Mumps incidences (cases/100,000 persons) per 5-years interval. In Western Germany notification of mumps started in 2013 and in Eastern Germany in 2001. Data were obtained from the Survstat tool of the German Robert-Koch-Institute (RKI) [74]. Data were accessed on 12 April 2018. Eastern Germany comprised Brandenburg, Mecklenburg-Western Pomerania, Saxony, Saxony-Anhalt and Thuringia. The other 11 federal states were regarded as Western Germany.

In 2002 the median age of infected patients in Eastern Germany was 11 years. In 2016, the median age of mumps patients in that region was 33 years (Figure 4). As vaccination against mumps had been initiated only eleven years earlier, this is, to the best of our knowledge, the first proof that initiation of mumps vaccination increases the age of affected patients in a population in which no outbreaks had occurred. In 2016 the median age of mumps infected individuals in Western Germany (25 years) was markedly lower than in Eastern Germany (33 years). In contrast to the Eastern part in Western Germany various outbreaks had been reported and the median age of affected patients was 16 to 24 years explaining the lower age of affected patients in Western Germany [75].

The largest outbreak was noticed in Bavaria (N = 295) lasting from 2010 to 2011 [38,76]. However, due to the results of serological analyses, the real number of affected patients was probably much higher [77]. In 2016, 34 infections were reported to Bavarian health authorities. Also in that year, the real number of affected individuals should have been higher since several local outbreaks were reported by daily papers. 

Apart from notifications to the health authority, information about the incidence of mumps, summarizing the outcome of laboratory tests were published in the German Reference Center for Measles, Mumps and Rubella (NRC MMR) at the RKI. The NRC MMR receives patients’ samples from physicians and public health agencies to confirm mumps diagnosis. From 2008 to 2013 samples from 534 patients had been submitted and MuV infection was confirmed for 216 patients. The majority of these patients lived in Western Germany (N = 530), e.g., in Bavaria (N = 134) and Lower Saxony (N = 44). Information about the vaccination status of 87 patients was available and 23 had not been vaccinated. Median age of these patients was 26.4 years, and 65% were 15–24 year old. Therefore, these data match those obtained from the notification data bank. Due to the information given by the physicians, who had submitted the samples to the NRC MMR, regional outbreaks in Bavaria and Lower Saxony were observed [77].

## 6. Conclusions

The present study shows that vaccination against mumps was successful in reducing its incidence, although outbreaks occurred in various countries and eradication of the disease did not happen. The main reason for this is waning immunity, while genetic divergences of vaccine and wild-type strains are a matter of discussion.

In Germany, vaccination against mumps resulted in a marked decrease in incidence. In Western Germany, incidence was higher due to local/regional outbreaks. In Eastern Germany, incidence was lower, possibly due to higher vaccination rates. Although the initiation of the vaccination campaign resulted in a low incidence, the age of affected individuals increased, as shown by an analysis of Eastern German notification data. As the probability to suffer from complications increases in older individuals, lack of vaccination increases the risk of complications over-proportionally, when living in a vaccinated population.

Although the benefit of a third MMR dose is temporally limited the authors feel that application of a third dose to adolescents might be appropriate to prevent mumps outbreaks since young adults become affected the most. As shown before, in Germany the second MMR vaccine is applied early in childhood (age of 15–23 months). Therefore, in Germany this measure might be more efficient compared to countries in which individuals are vaccinated later in live.

## Figures and Tables

**Table 1:** Measles, mumps, rubella (German pox) (MMR) and measles, mumps, rubella, varicella (chicken pox) vaccines (MMRV) available in Germany.

Name	Manufacturer	Viruses	Mumps Virus Strain	Dosage (TCID_50_)	Costs (Germany)
MMR VAXPRO	MSD *	MMR	Jeryl Lynn	>20,000	48.79 Euro
Proquad	MSD *	MMRV	Jeryl Lynn	>20,000	103.28 Euro
Priorix	GSK **	MMR	RIT 4385	>25,000	51.30 Euro
Priorix Tetra	GSK **	MMRV	RIT 4385	>25,000	103.47 Euro
MMR II	MSD *	MMR	Jeryl Lynn	>12,500	
M-M-RvaxPro	MSD *	MMR	Jeryl Lynn	>12,500	
Tresivac	Serum Institute of India Ltd.	MMR	L-Zagreb	>5000	96.25 INR *** (≈1.19 Euro)

TCID_50_ = Tissue Culture Infection Dose 50. * Merck Sharp & Dohme. ** GlaxoSmithKline. *** Costs in India according to [25]. Data were accessed on 16 April 2018. Tresivac is only available if no vaccines approved for use in Germany can be supplied (§73(3) Medicinal Products Act in the version published on 12 December 2005 (Federal Law Gazette [BGBl.]) Part I p. 3394, last amended by Article 3 of the Law of 4 April 2016 (Federal Law Gazette I p. 569). INR = Indian Rupee. On 16 April 1 Euro was about 81 Indian Rupee.

**Table 2:** Studies indicating waning immunity as a major risk factor of mumps in the MMR vaccination era. The studies were obtained through a “pubmed” search using the terms “mumps” AND “vaccination” AND “waning immunity” [42]. Data were accessed on 24 March 2018.

Study	Observation				
Rubin et al., 2008 [9]	Sera of 88 children who had received their first MMR vaccine between 12 and 24 months and their second vaccine between 4 and 6 years of age had been examined. Sera for examination of MuV antibody titers (GMT) were collected a few days before and one month after the second vaccination and 10 years later when the children were 14–16 years of age. In response to the second vaccination the GMT of neutralizing antibodies to 2 different MuV viruses significantly increased by 2.6-fold and 2.0-fold, respectively. 10 years later level of neutralizing antibodies was similar to that observed before the second MMR application.				
Lewnard et al., 2017 [14]	In a meta-analysis it was calculated that immunity after receipt of any mumps vaccine persists on average 27.4 years (CI 16.7–51.1 years). Among vaccinated individuals 25% may lose protection within 7.9 years (CI: 4.7–14.7 years), 50% within 19.0 years (11.2–35.4 years), and 75% within 38.0 years (22.4–70.8 years).				
Davidkin et al., 2008 [39]	The authors examined children after receipt of the second MMR. 20 years after vaccination only 40% of the children exhibited an antibody level interpreted as positive, 34% were equivocal and 26% were negative. Within 8 and 15 years after vaccination GMT decreased from 1:2491 to 1:767 to 1:597, respectively. The decline was more pronounced in males than in females.				
Cortese et al., 2008 [40]	In an outbreak at a college in Kansas (USA) students vaccinated twice had been examined. Those who had received their second dose 10 years before the outbreak or earlier more likely got affected (OR 2.46, 95% CI: 1.25–4.82)				
Seagle et al., 2018 [43]	From children having received the second MMR GMT were determined in an observation period of up to 12 years. Decline of GMT was 9.2% per year.				
LeBaron et al., 2009 [44]	Children received the second MMR either at kindergarten or middle-school entry. Although the response to the vaccine was vigorous 12 years later antibody titers were similar to those measured before the second MMR.				
Briss et al., 1994 [45]	In an outbreak at a high school in Tennessee RR was 2.9 (95% CI: 0.7–11.6) for students vaccinated before 1988 in comparison to those vaccinated later.				
Hersh et al., 1991 [46]	Students vaccinated 4 years before an outbreak in Kansas (USA) had a higher attack rate than those vaccinated more recently (RR = 5.2, 95% CI: 0.6–30).				
Schwarz et al., 2010 [47]	In an outbreak in Moldovia VE of 1 dose vaccination declined from 91% (95% CI: 88–92%) in 2-year-olds to 72% (95% CI: 70–74%) in 15- to 19-year-olds.				
Vygen et al., 2016 [48]	In various mumps clusters in France the odds of mumps significantly increased for individuals vaccinated twice by 10% for every year that had passed since the second dose (aOR 1.10; 95% CI: 1.02–1.19).				
Cohen et al., 2007 [49]	In England VE of 1/2 doses vaccination decreased with older age of children. VE of 1 dose vaccination in 2-year-olds: 96% (95% CI: 81–99%) VE of 1 dose vaccination in 11–12-year olds: 66% (95% CI: 30–83%) VE of 2 doses vaccination in 5–6-year-olds: 99% (95% CI: 97–99.5%) VE of 2 doses vaccination in 11–12-year olds: 86% (95% CI: 74–93%)				
Braeye et al., 2014 [50]	In an outbreak at a university in Flanders (Belgium) risk of students vaccinated twice was examined. Those who had been vaccinated 10 years ago or less had a lower risk than students vaccinated more than 10 years ago (RR: 0.33, 95% CI: 0.10–1.02).				
Man et al., 2012 [51]	VE for a single dose of mumps vaccine was 65% (95% CI: 19–85%) when applied within the past 3 years 15% (95% CI: −2–25%) when applied 3–6 years before an outbreak in Chinese schools.				
Castilla et al., 2009 [52]	In an outbreak in Spain affecting children older than 15 months risk of children who had received 2 vaccine doses was higher for children who had received the second dose 3 or more years before study enrollment (OR = 10.2, 95% CI: 1.5–70.7).				
Cardemil et al., 2017 [53]	Attack rates of students during a mumps outbreak vaccinated twice depended on the time since the second vaccination:				
	Time passed since the 2nd vaccination (years)	≤2	3–12	13–15	16–23
	Attack rate per 1000 population	1.6	3.9	11.3	17.6

VE = Vaccine effectiveness, CI = Confidence interval, RR = Relative risk, OR = Odds ratio, aOR = Adjusted odds ratio, GMT = Geometric mean titre.
